# New findings on the early life stages of five codlet species (Teleostei, Gadiformes, Bregmacerotidae) from the northwestern Pacific, including a putative undescribed *Bregmaceros* lineage

**DOI:** 10.3897/zookeys.1284.191403

**Published:** 2026-07-10

**Authors:** Seo-Yeon Koo, Se Hun Myoung, Sang-Chul Yoon, Jung-Hoon Lee, Jin-Koo Kim

**Affiliations:** 1 Department of Marine Biology, Pukyong National University, Busan 48513, Republic of Korea Department of Marine Biology, Pukyong National University Busan Republic of Korea; 2 Fisheries Resources Research Center, National Institute of Fisheries Science, Tongyeong 53064, Republic of Korea Fisheries Resources Research Center, National Institute of Fisheries Science Tongyeong Republic of Korea

**Keywords:** Codlet, identification key, juvenile, larvae, Northwestern Pacific, taxonomy

## Abstract

Codlets (Bregmacerotidae) play a major role in marine ecology, acting as trophic links between primary consumers and higher predators in mesopelagic communities; however, little information has been accumulated on their early life stages. In this study, 53 larval and juvenile specimens of Bregmacerotidae, collected from the northwestern Pacific Ocean between May 2017 and July 2024, were examined using 390-bp of mitochondrial DNA (mtDNA) 16S rRNA sequence analysis for molecular species identification. Five codlet species were identified: *Bregmaceros anchovia* (*n* = 1), *B. japonicus* (2), *B. nectabanus* (4), *B. neonectabanus* (6), and *Bregmaceros* sp. (20), a candidate undescribed species closely related to *B. atlanticus*. It was also discovered that previously recorded codlet larvae and juveniles appear to have been misidentified due to extreme similarity among external traits. Diagnostic characters enabling species identification at different developmental stages included the presence of melanophores on the lower right side of the preopercle, the pattern of lateral body melanophores, the presence of melanophores on the caudal peduncle, and the degree of first dorsal fin development. Here, we provide morphological descriptions and diagnostic keys for larvae and juveniles of these five taxa and hypothesize the existence of a putative undescribed *Bregmaceros* lineage in the region.

## Introduction

Codlets in family Bregmacerotidae comprise a single genus, *Bregmaceros*, with 16 species known worldwide (Harold and Baltzegar 2023), of which six have been reported in Japan (Motomura 2020) and five have been reported from Korea (Kang et al. 2025; Koo and Kim 2026). Bregmacerotids are distributed across temperate to tropical regions, occurring from surface waters to the mesopelagic zone at depths of up to 1000 m, although some species are also found in coastal areas and estuaries (Cohen et al. 1990; Nelson et al. 2016). Codlets play important ecological roles within marine environments, acting as trophic links between primary consumers and larger predator species in mesopelagic communities via extensive vertical migration (Matsuura et al. 1993; Zavala-García and Flores-Coto 1994). Previous studies on the Bregmacerotidae have focused primarily on adult fishes, including descriptions of new species, redescriptions of recorded species, fossil records, and osteological analyses (Dean and Houde 1984; Masuda et al. 1986; Świdnicki 1991; Torii et al. 2003; Ho et al. 2020a, 2020b; Ekin 2022; Harold and Baltzegar 2023). In contrast, research on their early life history has been relatively limited. Houde (1984) described the characteristics of bregmacerotid eggs, while Munro (1950) first described the larvae of three species (*Bregmaceros macclellandii*, *Bregmaceros japonicus*, and *Bregmaceros nectabanus*) collected from Australian waters. Later, Namiki et al. (2007) reported three larval species from the southwestern Atlantic Ocean (*Bregmaceros atlanticus*, *Bregmaceros cantori*, and *Bregmaceros* sp. nov.). Okiyama (2014) described the larvae of *B. japonicus*, *B. nectabanus*, *Bregmaceros neonectabanus*, and an undescribed species from Japanese waters. Although larval occurrences of several species have since been reported, many remain undescribed or are known only from limited developmental stages, resulting in a paucity of information on bregmacerotid larvae (Houde 1981; Mamhot et al. 1992; Matsuura et al. 1993). Previous studies on larval fishes from Korean waters have reported that larvae of various fish groups, including Engraulidae and Salangidae, are frequently difficult to distinguish morphologically from closely related species during early developmental stages (Koo et al. 2024; Ryu et al. 2024). However, these taxa were clearly distinguished using molecular markers, and subsequent detailed morphological analyses revealed diagnostic characters, including melanophore patterns (Koo et al. 2024; Ryu et al. 2024). The lack of taxonomic information and reliable identification keys for larval fishes has limited our understanding of nursery grounds and early life-history characteristics, thereby hindering efficient fisheries resource management. Furthermore, melanophore patterns in larval Bregmacerotidae can vary considerably depending on developmental stage and preservation condition, resulting in many species still lacking proper morphological descriptions corroborated by molecular data. Therefore, integrating molecular data with morphological observations is essential for accurate species identification and clarification of taxonomic relationships among closely related taxa.

Information on the early life history of marine fishes provides valuable insights into phylogenetic relationships based on ontogenetic morphological variations and can be used to interpret geographic distribution patterns (Yamaguchi et al. 2000; Roje 2010; Gill and Leis 2019). Such information is also essential for identifying spawning grounds and seasons, estimating stock abundance, and supporting accurate species identification (Miller and Kendall 2009). Therefore, this study was performed to identify bregmacerotid larvae collected over an 8-year period (2017–2024) from the northwestern Pacific, including Korean waters, by integrating morphological and molecular approaches. Species identification at the molecular level was conducted by mitochondrial DNA (mtDNA) 16S rRNA sequence analysis in comparison with adult voucher specimens (Kochzius et al. 2010). The morphological traits of each developmental stage were compared in detail with those described in previous studies (Mamhot et al. 1992; Yoo et al. 1992; Okiyama 2014) to confirm misidentification, diagnostic characteristics were established, and a larval identification key was developed for the taxa we evaluated. In addition, potential cryptic diversity in larval fishes is discussed along with our hypothesis of a putative undescribed bregmacerotid species in Korean waters based on our results. Accurate identification of larval Bregmacerotidae is important for understanding species diversity, spawning ecology, and early life-history characteristics in the northwestern Pacific Ocean. The present study also provides baseline information for future taxonomic, ecological, and biogeographic studies of larval fishes in Korean waters.

## Materials and methods

### Sample collection

**Figure 1.**
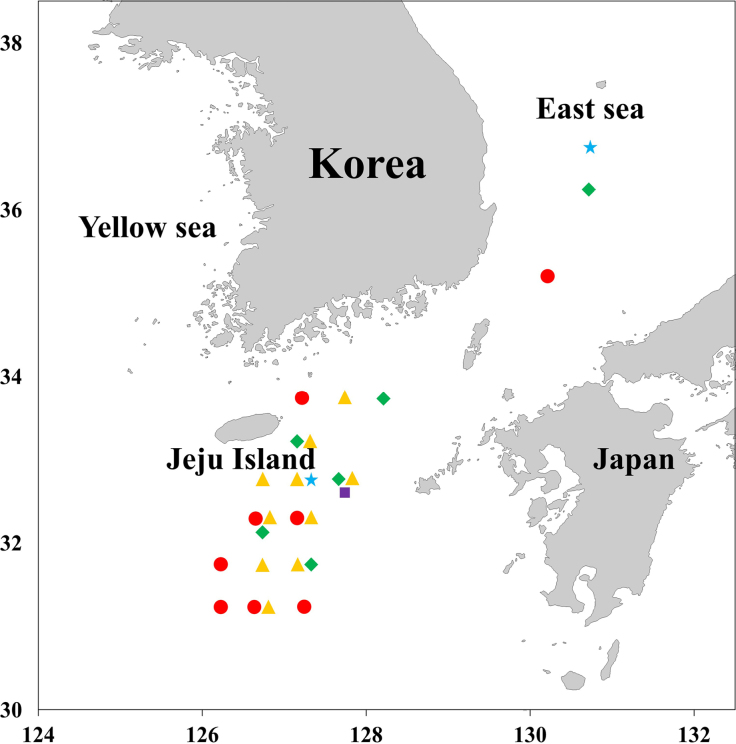
Sampling area for Family Bregmacerotidae larvae and juveniles: *Bregmaceros japonicus* (blue star); *Bregmaceros nectabanus* (red circle); *Bregmaceros neonectabanus* (green diamond); *Bregmaceros anchovia* (purple square); ‘*Bregmaceros* sp.’ (yellow triangle).

A total of 53 larval codlet specimens were examined. Specimens were collected from the northwestern Pacific Ocean, including the East Sea, Korea Strait, and adjacent waters of Jeju-do Island, between May 2017 and July 2024 during trips on research vessels (TAMGU 21-23) belonging to the National Institute of Fisheries Science (NIFS) (Fig. 1). Zooplankton samples were collected aboard a research vessel using bongo nets (mouth opening: 80 cm; mesh size: 500 μm). The cruising speed was 2–3 knots, and oblique tows were conducted during daytime hours (09:00–18:00) for 10 min at each site from depths of approximately 80–130 m to the surface. Larval fishes sorted from the zooplankton samples were immediately fixed in 10% buffered formalin for 30 min, rinsed, and subsequently preserved in 99% ethanol. Larval fishes were identified to the lowest possible taxonomic level based on morphological characters following Okiyama (2014). All specimens were deposited in the Ichthyoplankton Laboratory, Pukyong National University (**PKUI**), and catalogued as PKUI 1297–1350 (Table 1). To confirm species-level identification of the codlet larvae collected in the present study, we developed a reference library based on adult voucher specimens and their mtDNA16S rRNA sequences. Adult specimens and muscle tissues were examined or obtained on loan from the following institutions (Table 2): Kagoshima University Museum (**KAUM**), Virginia Institute of Marine Science (**VIMS**), Kanagawa Prefectural Museum of Natural History (**KPM-NI**), Academia Sinica Institute of Zoology (**ASIZP**), Texas A&M University (**TCWC**).

**Table 1.** Voucher number, collection information, and GenBank number for five species of the family Bregmacerotidae in present study.

**Species**	***n***	**Sampling month**	**Locality**	**Voucher number**	**Stage**	**GenBank #**
***Bregmaceros japonicus***	2	5	East sea, Jeju-do island	PKUI 1297-1298	Postflexion larva – Juvenile	PX457549–PX457550
***B. nectabanus***	24	7–11	East sea, Jeju-do island	PKUI 1299-1322	Preflexion larva – Juvenile	PX457527–PX457547
***B. neonectabanus***	6	9–11	East sea, Jeju-do island	PKUI 1323-1328	Preflexion larva, Postflexion larva, Juvenile	PX457420–PX457423
***B. anchovia***	1	8	Jeju-do island	PKUI 1329	Juvenile	PX457548
***Bregmaceros* sp**.	20	3–11	Jeju-do island	PKUI 1330-1349	Preflexion larva – Juvenile	PX457551–PX457563

**Table 2.** List of comparative specimens or muscle tissues loaned from various research institutes used in this study.

**Species**	**Institution**	**Locality**	**Voucher number**
***B. neonectabanus***	Kagoshima University Museum	Kagoshima, Japan	KAUM-I.104814
***B. neonectabanus***	Kagoshima University Museum	Kagoshima, Japan	KAUM-I.111890
***B. atlanticus***	Virginia Institute of Marine Science	Atlantic Ocean, Commonwealth of The Bahamas	VIMS 5829
***B. atlanticus***	Virginia Institute of Marine Science	Center of Eddy C2	VIMS 36811-1
***B. atlanticus***	Virginia Institute of Marine Science	Center of Eddy C2	VIMS 36811-2
***B. anchovia***	Kanagawa Prefectural Museum of Natural History	Miyazaki, Japan	KPM-NI0062323
***B. pseudolanceolatus***	Kanagawa Prefectural Museum of Natural History	Dong-gang, Taiwan	KPM-NI0048150
***B. cantori***	Texas A&M University	Gulf of Mexico	TCWC 19204.01
***B. cantori***	Texas A&M University	Gulf of Mexico	TCWC 19208.01
***B. lanceolatus***	Academia Sinica Institute of Zoology	Dong-gang, Taiwan	ASIZP0065753
***B. pseudolanceolatus***	Academia Sinica Institute of Zoology	Dong-gang, Taiwan	ASIZP0074795

### Molecular analysis

Genomic DNA was extracted from the muscle tissues of seven adult fishes and from the right eyeball of 53 larvae and juveniles using AccuPrep® Genomic DNA Extraction Kit (Bioneer, Daejeon, Korea). Extracted DNA was stored at −18 °C until used in molecular analyses.

Mitochondrial DNA 16S ribosomal RNA (16S rRNA) was amplified using the polymerase chain reaction (PCR) with following universal primers 16Sar (5’-CGC CTG TTT ATC AAA AAC AT-3’) and 16Sbr (5’-CCG GTC TGA ACT CAG ATC AGG T-3’; Palumbi 1996). PCR reactions were performed in a total volume of 20 μL and contained 2 μL of DNA template, 2 μL of 10× PCR buffer, 1.6 μL of 2.5 mM dNTPs, 0.5 μL of each primer, 0.1 μL of TaKaRa EX-Taq polymerase, and distilled water to volume. PCR was carried out using Bio-Rad T-100 thermocycler (Bio-Rad, Hercules, California, USA) under the following conditions: initial denaturation at 95 °C for 5 min; 31 cycles of denaturation at 94 °C for 30 s, annealing at 56–58 °C for 30 s (56 °C for *Bregmaceros neonectabanus* and 58 °C for the remaining species), extension at 72 °C for 30 s, followed by final extension at 72 °C for 5 min. Sequences were obtained by cycle sequencing using a BigDye Terminator v. 3.1 Cycle Sequencing Kit (Applied Biosystems, Foster City, CA, USA) with ABI Prism 3730XL analyzer (Applied Biosystems, Foster City, CA, USA).

Obtained 16S rRNA sequences were compared with reference sequences available in the National Center for Biotechnology Information (NCBI) database using the Basic Local Alignment Search Tool (BLAST). Specimens showing ≥ 99% sequence similarity to reference sequences were considered conspecific (Kim et al. 2014). Resulting 16S rRNA sequences were aligned using ClustalW (Thompson et al. 1994) in BioEdit software (v. 7.2.5; Hall 1999). Genetic distances were calculated using the Kimura 2-parameter (K2P) model (Kimura 1980) in MEGA XI software (Tamura et al. 2021). The K2P model was used because previous studies have shown that differences between K2P and best-fit model distance estimates are generally minimal for species identification purposes (Collins et al. 2012). In the present study, genetic distances were primarily used for species-level comparison and barcode-based discrimination rather than for evolutionary rate estimation. Therefore, the use of K2P distances was considered appropriate for the objectives of this study and also facilitated comparisons with previous DNA barcoding studies of fishes. Phylogenetic relationships were inferred using the maximum likelihood (ML) method as implemented in MEGA XI (Tamura et al. 2021) based on combined sequences of adult and larval specimens. The best-fit substitution model (GTR+G) was selected using jModelTest 2.1.10 (Darriba et al. 2012) based on the Akaike Information Criterion (AIC). Confidence in ML tree patterns of relationship was evaluated by conducting 1,000 bootstrap pseudoreplicates in MEGA XI (Tamura et al. 2021) and bootstrap proportions were annotated along the final ML tree branches. Sequence data of other species in the genus *Bregmaceros* were obtained from the NCBI Nucleotide database (National Library of Medicine, Bethesda, Maryland, USA) and included: *Bregmaceros japonicus* (MZ182269), *Bregmaceros nectabanus* (MZ182261), *Bregmaceros atlanticus* (FJ215104), *Bregmaceros cantori* (FJ215105, PX457525, PX457526), *Bregmaceros neonectabanus* (PX457418, PX457419), *Bregmaceros lanceolatus* (PX457524), and *Enchelyopus cimbrius* (KC980958) as the outgroup.

### Morphological analysis

**Figure 2.**
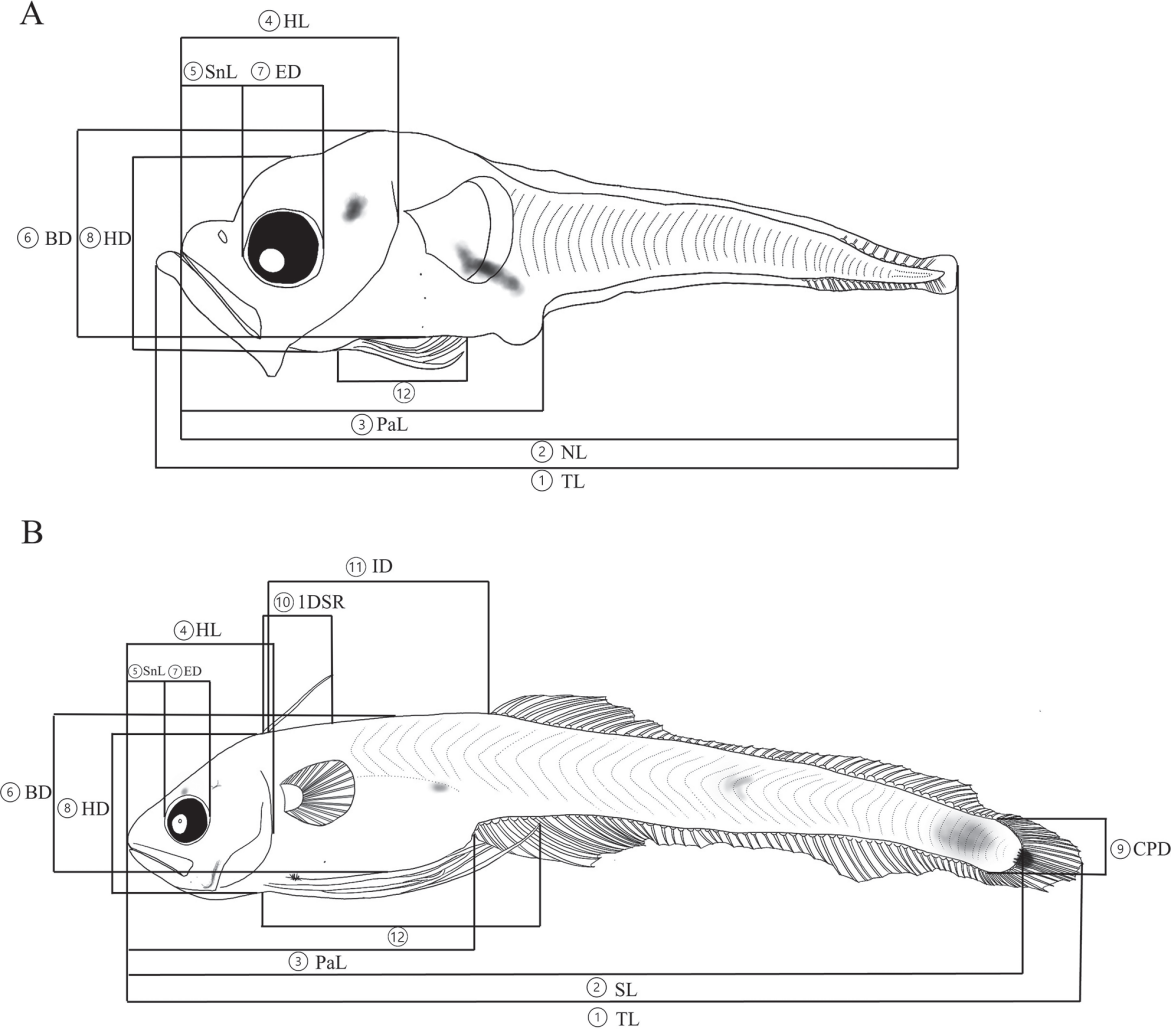
The measurements of Family Bregmacerotidae. **A**. Larvae; **B**. Juveniles. 1, Total length (TL); 2, Notochord length (NL), Standard length (SL); 3, Pre-anal length (PaL); 4, Head length (HL); 5, Snout length (SnL); 6, Body depth (BD); 7, Eye diameter (ED); 8, Head depth (HD); b-9, Caudal peduncle depth (CPD); b-10, 1^st^ dorsal soft ray length (1DSR); b-11, Interdorsal distance (ID); 12, Longest pelvic fin ray length.

Terminology used for body parts and developmental stages in this study followed that of Okiyama (2014) and Ji et al. (2020). Taxonomic nomenclature and classification followed Eschmeyer’s Catalog of Fishes (Fricke et al. 2026). Meristic characters examined included counts of dorsal fin rays (**D**), pectoral fin rays (**P1**), pelvic rays (**P2**), anal fin rays (**A**), and caudal fin rays (**C**). Caudal fin rays were counted as branched plus unbranched rays following Masuda et al. (1986). Morphometric measurements included total length (**TL**), notochord length (**NL**) or standard length (**SL**), pre-anal length (**PaL**), head length (**HL**), snout length (**SnL**), body depth (**BD**), eye diameter (**ED**), head depth (**HD**), caudal peduncle depth (**CPD**), first dorsal soft ray length (**1DSR**), interdorsal distance (**ID**), Longest pelvic fin ray length (Fig. 2). Morphological characteristics were examined using a stereomicroscope (SZH-16; Olympus, Tokyo, Japan) and measurements were taken to the nearest 0.01 mm using Active measure program (Mosaic 2.0; Fuzhou Tucsen photonics, Fuzhou, China). Morphometric values are expressed as percentages (%) of notochord length (NL) or standard length (SL) and head length (HL). One representative specimen per developmental stage is illustrated for each species.

### Data resources

The data underpinning the analysis reported in this paper are deposited at GBIF, the Global Biodiversity Information Facility, and are available at https://doi.org/10.15468/w7kabz.

## Results

### Molecular identification

In total, we analyzed 53 larval specimens of family Bregmacerotidae. Amplification of a 390-bp mtDNA16S rRNA sequence region, followed by sequence comparison and ML analysis, identified five taxa among these specimens, as follows: *Bregmaceros japonicus* (PX457549–PX457550), *Bregmaceros nectabanus* (PX457527–PX457547), *Bregmaceros neonectabanus* (PX457420–PX457423), *Bregmaceros anchovia* (PX457548), and a putative undescribed form informally referred to herein as, ‘*Bregmaceros* sp.’ (PX457551–PX457563). The intraspecific genetic distances among these taxa ranged from 0% to 0.66%, indicating a high degree of genetic similarity within species. The interspecific genetic distances among the five identified taxa collected in the present study ranged from 7.34% to 23.40% (Table 3), indicating substantial genetic divergence between taxa, including the described and undescribed forms. The lower genetic distance (5.93%) was obtained from the comparison between *Bregmaceros* sp. and reference *B. atlanticus* sequences retrieved from GenBank.

**Table 3.** Mean genetic distances among five species of family Bregmacerotidae based on mtDNA16S rRNA sequences.

	**1**	**2**	**3**	**4**	**5**
***Bregmaceros nectabanus***	0.000				
***Bregmaceros neonectabanus***	0.114	0.000			
***Bregmaceros* sp**.	0.188	0.151	0.000		
***Bregmaceros japonicus***	0.185	0.170	0.098	0.000	
***Bregmaceros anchovia***	0.073	0.147	0.234	0.193	0.000

**Figure 3.**
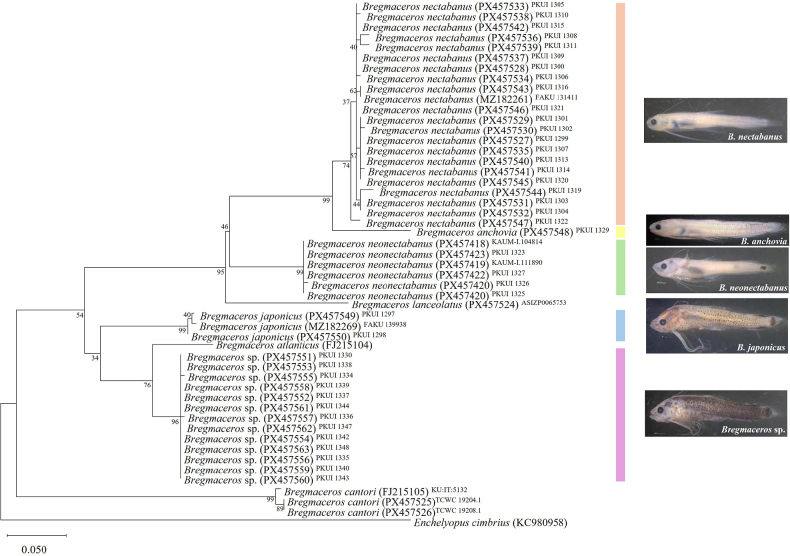
Maximum-likelihood (ML) tree showing the phylogenetic relationships of family Bregmacerotidae based on mtDNA16S rRNA region. Scale bar indicates genetic distances of 0.050. The values at the nodes indicate bootstrap values.

In the ML tree, *B. cantori* formed a sister group to all other congeners, representing the earliest-diverging lineage within the genus. The identified taxa were recovered as distinct clades, with *B. japonicus*, *B. neonectabanus*, and *Bregmaceros* sp. strongly supported by bootstrap values of 99%, 99%, and 96%, respectively. Additionally, *B. japonicus*, *B. atlanticus*, and *Bregmaceros* sp. formed a sister group to a clade containing *B. nectabanus*, *B. anchovia*, *B. neonectabanus*, and *B. lanceolatus*. Within the three-lineage clade, *Bregmaceros* sp. was recovered as a sister lineage to *B. atlanticus* supported by bootstrap values of 76%, suggesting a close relationship between these two species/lineages. *B. lanceolatus* formed a sister group to a clade containing *B. neonectabanus*, *B. anchovia*, and *B. nectabanus*. Among these, *B. neonectabanus* was sister to a clade comprised of *B. nectabanus* + *B. anchovia*, thus *B. anchovia* was strongly supported as the sister lineage to *B. nectabanus* tips, indicating a close evolutionary relationship between these two species (Fig. 3).

### Diagnostic characters of Bregmacerotidae larvae

#### Systematics accounts

**Family Bregmacerotidae Gill, 1872 (Korean name: Nal-gae-myeol-gwa)**

##### Genus Bregmaceros

Taxon classificationAnimaliaGadiformesBregmacerotidae

Thompson, 1840

C1B8D7C0-2650-5997-9145-996B1866980A

###### Korean name.

Nal-gae-myeol-sok.

###### Type species.

*Bregmaceros mcclellandi* Thompson, 1840.

###### Description.

Body elongate, laterally compressed, tapering posteriorly; elongated first dorsal fin ray and pelvic fin ray; second dorsal fin and anal fin rays with concave mid region.

##### Bregmaceros anchovia

Taxon classificationAnimaliaGadiformesBregmacerotidae

Ho, Endo & Lee, 2020

3B060651-BC35-53DA-B1EC-066163BA4EE2

###### Korean name.

Song-got-ip-nal-gae-myeol.

###### Description.

**Figure 4.**
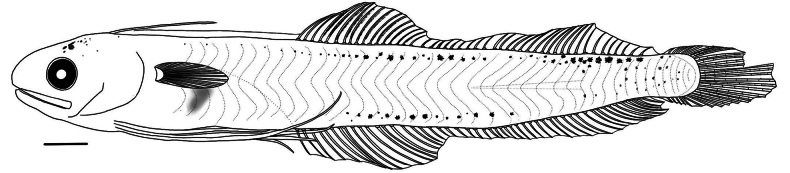
Illustration of juvenile of *Bregmaceros anchovia*. Juvenile (PKUI 1329, 16.18 mm SL). Scale bar: 1.000 mm.

**Juvenile** (16.18 mm SL). (Fig. 4; Table 4).

**Table 4.** Measurements and counts of juvenile of *Bregmaceros anchovia*.

**Species**	***Bregmaceros anchovia***
Stage	**Juvenile**
Number of specimens	1
**Measurements**	
Total length (mm, TL)	18.07
Standard length (mm, SL)	16.18
% in SL	
Head length (mm, HL)	18.97
Pre-anal length (PaL)	41.01
Body depth (BD)	13.92
Head depth (HD)	13.35
Caudal peduncle depth (CPD)	6.14
1^st^ dorsal soft ray length (1DSR)	15.08
Interdorsal distance (ID)	27.46
Longest pelvic fin ray length	37.31
% in HL	
Snout length (SnL)	26.86
Eye diameter	21.71
**Counts**	
Dorsal-fin rays	1–47
Anal-fin rays	50
Pectoral-fin rays	17
Pelvic-fin rays	6
Caudal-fin rays	21+10

**Figure 5.**
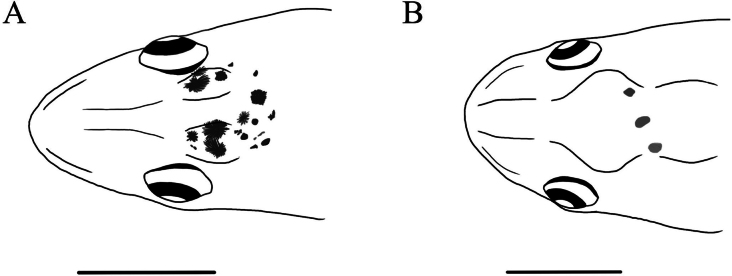
Illustration showing dorsal view of head. **A**. *Bregmaceros anchovia*; **B**. *Bregmaceros nectabanus*. Scale bars: 1.000 mm.

Body long, slightly compressed, tapering posteriorly. Anus anterior to mid-body. Snout slightly inclined upwards. Upper jaw projecting beyond lower; snout pointed in dorsal view (Fig. 5). Maxilla extending beyond center of pupil but not reaching posterior margin. All fin rays fully formed. First dorsal fin ray on the occipital region elongating further. Pectoral fin originating just posterior to operculum; located slightly above the mid-body depth. Pelvic fin elongating with growth. Longest ray extending well beyond anal fin origin. Anal fin origin immediately posterior to anus; second dorsal fin origin posterior to vertical through anal fin origin. Anterior and posterior rays of second dorsal and anal fins longer than middle rays, forming distinct concave profile. Caudal fin rays clearly divided into branched and unbranched rays. Melanophores densely distributed on head, consisting of stellate, dot-like, branch-shaped. A single large embedded melanophore present at anterior region of abdominal cavity. Small dot-shaped melanophores forming a single row from nape to end of second dorsal fin; becoming larger and stellated in shape from second dorsal fin origin. Stellate melanophores forming a single row on ventral side of trunk; terminating around the middle of anal fin. Dot-like melanophores sporadic on caudal peduncle. Melanophores present on anterior second dorsal fin rays.

###### Diagnostic characters.

Single rows of melanophores present along both dorsal and ventral sides of trunk. Pointed snout.

###### Remarks.

Although the larval specimen of *B. anchovia* examined in the present study was collected prior to the publication of the first adult record from Korea by Koo and Kim (2026), its identification was completed later following comparative morphological and molecular analyses. Therefore, the present study represents the first larval record of *B. anchovia* from Korean waters. The occurrence of larval *B. anchovia* around Jeju-do Island suggests that the northwestern Pacific Ocean may serve as a potential spawning and nursery ground for the species.

##### Bregmaceros japonicus

Taxon classificationAnimaliaGadiformesBregmacerotidae

Tanaka, 1908

FBBF11E0-E9EE-5854-9BAC-3FB62563DD6F

###### Korean name.

Nal-gae-myeol.

###### Description.

**Figure 6.**
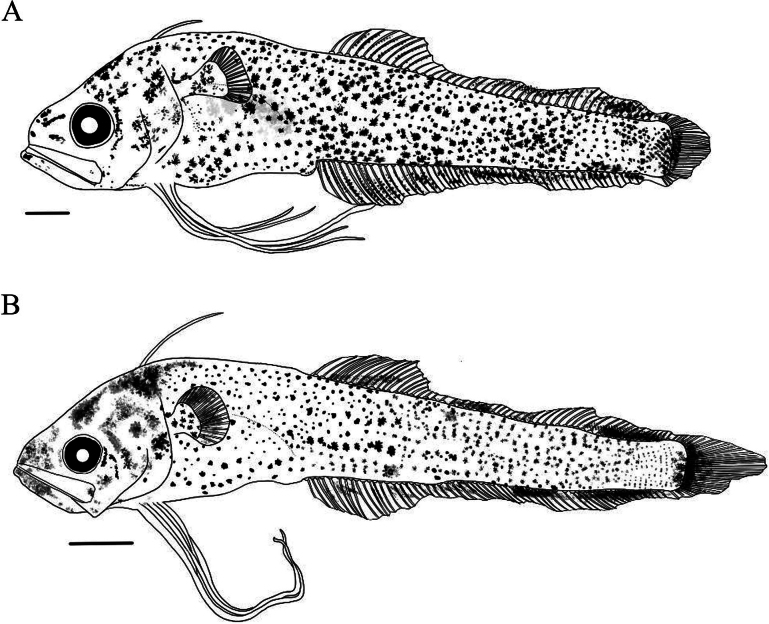
Illustrations of morphological development of *Bregmaceros japonicus*. **A**. Postflexion larva (PKUI 1297, 9.06 mm SL); **B**. Juvenile (PKUI 1298, 10.85 mm SL). Scale bars: 1.000 mm.

**Postflexion larva** (9.06 mm SL). (Fig. 6A; Table 5).

**Table 5.** Measurements and counts of larva and juvenile of *Bregmaceros japonicus*.

**Species**	***Bregmaceros japonicus***	
Stage	**Postflexion**	**Juvenile**
Number of specimens	1	1
**Measurements**		
Total length (mm, TL)	9.53	12.14
Standard length (mm, SL)	9.06	10.86
% in SL		
Head length (mm, HL)	27.71	23.56
Pre-anal length (PaL)	45.31	43.23
Body depth (BD)	22.22	20.39
Head depth (HD)	27.71	23.56
Caudal peduncle depth (CPD)	6.80	7.19
1^st^ dorsal soft ray length (1DSR)	14.26	13.89
Interdorsal distance (ID)	27.81	23.97
Longest pelvic fin ray length	33.55	42.78
% in HL		
Snout length (SnL)	24.54	30.53
Eye diameter	21.63	20.41
**Counts**		
Dorsal-fin rays	43	55
Anal-fin rays	50	57
Pectoral-fin rays	19	17
Pelvic-fin rays	6	6
Caudal-fin rays	28	20+10

Body elongate, laterally compressed, tapering posteriorly. Head depth exceeded body depth. Anus anterior to mid-body. Maxilla extending beyond center of pupil. First dorsal fin ray on the occipital region elongating further. Pelvic fin originating just posterior to lower jaw; longest ray extending beyond anal fin origin. Anal and second dorsal fins origin posterior to anus; anal fin origin slightly anterior to vertical through second dorsal fin origin. Anterior and posterior rays of second dorsal and anal fins elongate, with concave mid-region. Branch-shaped melanophores dense on head, jaws, snout, opercle, supraorbital region, and pectoral fin. Stellate melanophores scattered on the abdominal cavity, nape, and lateral trunk. Second dorsal and anal fin rays densely pigmented; melanophores clustered around caudal fin base.

**Juvenile** (10.86 mm SL). (Fig. 6B; Table 5).

Body long, slightly compressed, tapering posteriorly. Head depth exceeded the body depth. Anus anterior to mid-body. Maxilla extending beyond center of pupil but not reaching posterior margin. First dorsal fin ray on the occipital region elongating with growth. All fin rays fully formed. Pelvic fin elongating with growth. Longest ray extending well beyond anal fin base. Anal fin origin immediately posterior to anus; second dorsal fin origin posterior to vertical through it. Anterior and posterior rays of second dorsal and anal fins markedly longer than middle rays; forming distinct concave profile. Branch-shaped melanophores clustered on head, snout, jaws, preopercle. Pectoral fin base and rays densely pigmented. Stellate melanophores scattered on abdominal cavity, nape, and lateral trunk; the size of melanophores becoming smaller with growth. Scattered melanophores on second dorsal and anal fin rays, densest at posterior region. Caudal fin base heavily pigmented but not on rays.

###### Diagnostic characters.

Large melanophores distributed over entire body; melanophores densely clustered at bases of second dorsal, anal, and caudal fins.

##### Bregmaceros nectabanus

Taxon classificationAnimaliaGadiformesBregmacerotidae

Whitley, 1941

4DAF75BD-6DDD-5F4E-870E-4002CDD46EFC

###### Korean name.

Tae-pyong-yang-nal-gae-myeol.

###### Description.

**Figure 7.**
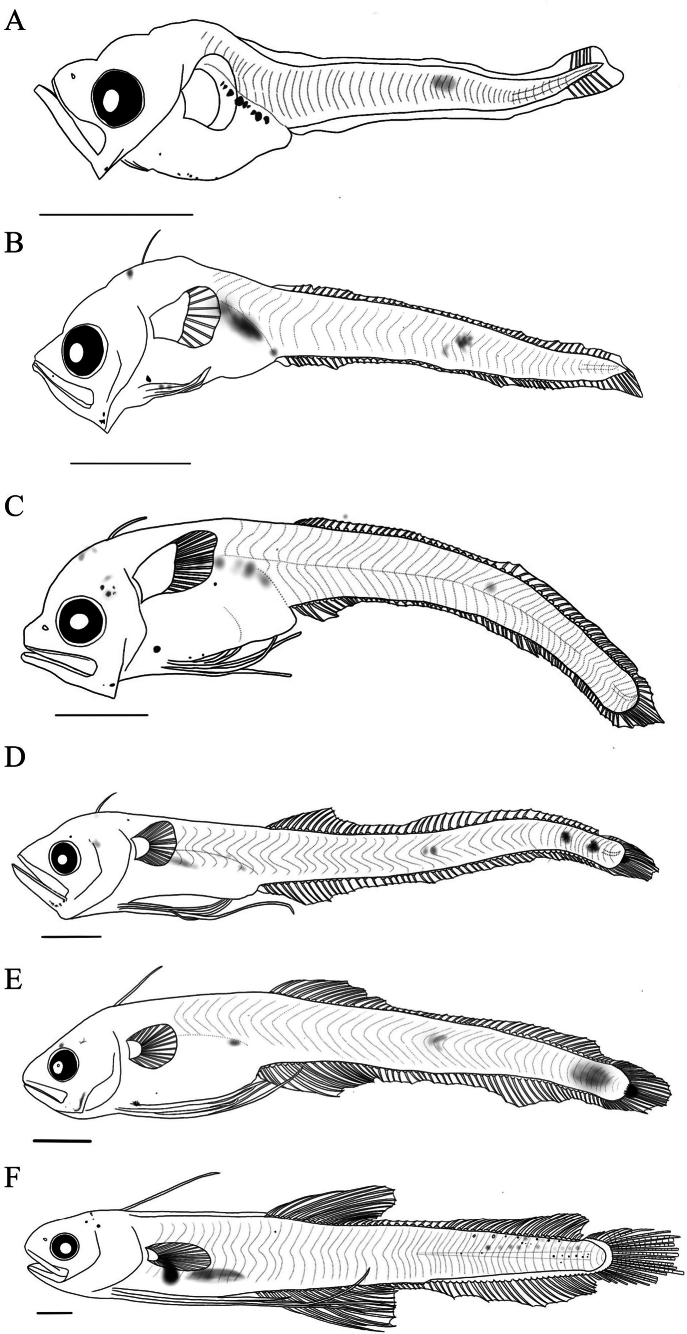
Illustrations of morphological development of *Bregmaceros nectabanus*. **A**. Preflexion larva (PKUI 1300, 3.66 mm NL); **B**. Flexion larva (PKUI 1303, 5.01 mm SL); **C**. Postflexion larva (PKUI 1316, 7.33 mm SL); **D**. Postflexion larva (PKUI 1306, 10.49 mm SL); **E**. Juvenile (PKUI 1299, 11.25 mm SL); **F**. Juvenile (PKUI 1312, 17.62 mm SL). Scale bars: 1.000 mm.

**Preflexion larvae** (2.81–4.03 mm NL). (Fig. 7A; Table 6).

**Table 6.** Measurements and counts of larvae and juveniles of *Bregmaceros nectabanus*.

**Species**	***Bregmaceros nectabanus***			
Stage	**Preflexion**	**Flexion**	**Postflexion**	**Juvenile**
Number of specimens	7	4	5	8
**Measurements**				
Total length (mm, TL)	2.82–4.29 (3.74)	4.50–5.34 (4.93)	6.88–11.28 (8.80)	11.00–35.94 (20.83)
Standard length (mm, SL)	2.81–4.03 (3.61)	4.36–5.04 (4.74)	6.69–10.49 (8.33)	10.23–31.52 (18.87)
% in SL				
Head length (mm, HL)	21.79–27.20 (24.42)	19.90–25.19 (23.10)	17.97–20.49 (18.85)	16.26–19.38 (17.61)
Pre-anal length (PaL)	42.92–48.56 (45.80)	39.23–45.74 (43.26)	36.91–40.24 (39.37)	37.81–39.46 (38.76)
Body depth (BD)	25.74–31.64 (28.36)	21.74–28.12 (24.94)	12.30–20.13 (17.29)	14.15–17.65 (15.52)
Head depth (HD)	24.85–31.86 (27.29)	19.98–26.87 (23.44)	16.41–19.64 (17.80)	12.86–16.01 (14.15)
Caudal peduncle depth (CPD)	0	0–6.19 (2.93)	4.48–6.49 (5.53)	5.07–6.19 (5.74)
1^st^ dorsal soft ray length (1DSR)	0	1.71–9.16 (6.02)	3.86–7.19 (5.61)	6.88–21.21 (15.37)
Interdorsal distance (ID)	0	20.75–44.58 (24.09)	23.54–27.03 (25.73)	19.41–29.82 (25.59)
Longest pelvic fin ray length	7.85–8.09 (7.97)	11.45–18.13 (14.35)	17.09–32.92 (24.95)	32.23–49.58 (43.10)
% in HL				
Snout length (SnL)	26.70–35.33 (30.93)	25.82–32.69 (29.94)	28.64–36.06 (33.01)	18.39–30.24 (23.79)
Eye diameter	31.41–42.19 (37.41)	29.22–34.15 (32.24)	22.61–32.91 (26.42)	16.74–25.75 (21.48)
**Counts**				
Dorsal-fin rays	0~6	1–21~42	1–48~53	1–48~55
Anal-fin rays	0~5	32~42	52~55	50~55
Pectoral-fin rays	0	8	8~15	16~19
Pelvic-fin rays	4~6	5~6	6	6
Caudal-fin rays	2	15~18	16~20	18~21+10~12

Body elongate, laterally compressed tapering posteriorly. Abdominal cavity rounded; anus anterior to mid-body. Head large, dorsally convex. Snout inclined upwards; lower jaw projecting beyond upper jaw. Posterior end of the maxilla not reaching the center of eye. First dorsal fin ray not formed. Pelvic fin bud present posterior to lower jaw. Pectoral fin as finfold. Anal fin origin immediately posterior to anus; second dorsal fin origin anterior to vertical through anal fin origin. Second dorsal and anal fins in most specimens as finfold; rays developing posteriorly in some specimens. Caudal fin continuous with second dorsal and anal fins; caudal rays forming beneath posterior notochord tip. Dot-like melanophores forming row on dorsal side of abdominal cavity. A few dot-like melanophores present on ventral side of abdominal cavity. A single dot-like melanophore on the lower operculum. A single melanophore on the mid-body.

**Flexion larvae** (4.36–5.04 mm NL). (Fig. 7B; Table 6).

Body compressed, elongate, tapering posteriorly. Head large with shallow dorsal depression. Abdominal cavity rounded; anus anterior to mid-body. Snout inclined; posterior tip of maxilla extending beyond center of pupil. Single elongate, separate first dorsal fin ray formed on the occipital region and reaching center of abdominal cavity. Pectoral fin developing rays; anterior portion formed pterygiophores. Anal fin origin immediately posterior to anus; second dorsal fin origin vertically above anal fin origin. A series of dot-like melanophores on the dorsal side of the abdominal cavity, decreasing with growth; but melanophores on isthmus becoming bigger and denser. Dot-like melanophores on lower operculum becoming more distinct. A melanophore on the lateral trunk, becoming slightly darker but smaller.

**Postflexion larvae** (6.69–10.49 mm SL). (Fig. 7C, D; Table 6).

Body elongate, laterally compressed, tapering posteriorly. Anus anterior to mid-body; abdominal cavity rounded. Snout less steep than in flexion larvae. Posterior tip of maxilla extending beyond center of pupil. First dorsal fin ray on the occipital region becoming longer. In smaller specimens (~7.33 mm SL), pelvic fin originating just posterior to lower jaw; longest ray extending beyond anus. In larger specimens (~10.49 mm SL), pelvic fin rays elongating further; longest ray extending beyond anal fin origin. Anal fin origin immediately posterior to anus; second dorsal fin origin slightly posterior to vertical through anal fin origin. In smaller specimens (~7.33 mm SL), second dorsal and anal fin rays similar in length; in larger specimens (~10.49 mm SL), anterior and posterior rays elongate, with concave mid-region. Embedded melanophores present at dorsal side of abdomen; a single melanophore on isthmus. Melanophores appearing on head and supraorbital region. Lower operculum heavily pigmented. In larger specimens (~10.49 mm SL), two large melanophores on posterior trunk, and a single melanophore on caudal fin base.

**Juveniles** (10.23–31.52 mm SL). (Fig. 7E, F; Table 6).

Body long, slightly compressed, tapering posteriorly. Anus anterior to mid-body. Snout slightly inclined upwards and rounded in dorsal view (Fig. 5). Maxilla reaching or slightly exceeding center of pupil. First dorsal fin ray on the occipital region elongating further. All fin rays fully formed. Pelvic fin elongating with growth; longest ray extending well beyond anal fin origin. Second dorsal fin origin slightly posterior to vertical through anal fin origin. Caudal fin rays divided into branched and unbranched rays. In smaller specimens (~11.27 mm SL), melanophore on dorsal side of abdominal cavity and lateral trunk almost absent; a single melanophore on ventral side of abdominal cavity remained. Supraorbital melanophores becoming weaker. Thin melanophores present at lower side of operculum. Melanophores on lateral trunk becoming a single large melanophore. Caudal fin base densely pigmented. However, in larger specimens (17.62 mm SL), large embedded melanophores present at abdominal cavity; dot-like melanophores on the head. Melanophores absent on the lower side of the operculum. Melanophores on posterior trunk absent; dot-like melanophores forming a single row dorsally. Melanophores on caudal fin rays absent.

###### Diagnostic characters.

Less pigmented larvae compared with *B. japonicus*. Melanophores present on lower part of preopercle.

##### Bregmaceros neonectabanus

Taxon classificationAnimaliaGadiformesBregmacerotidae

Masuda, Ozawa & Tabeta, 1986

FFA3806C-5E5E-52FE-92D0-2A24D027F445

###### Korean name.

Un-nal-gae-myeol.

###### Description.

**Figure 8.**
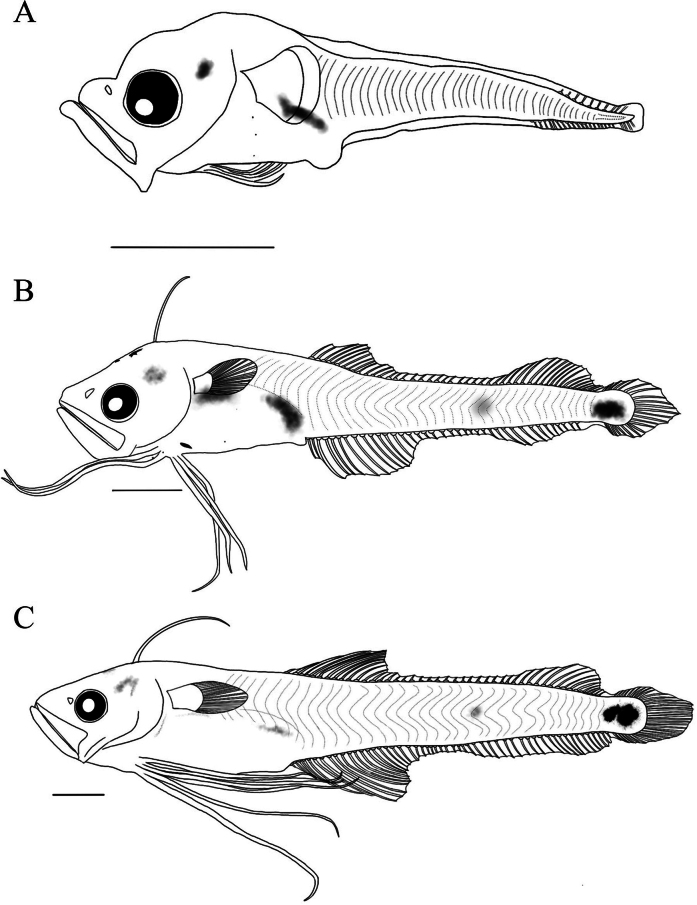
Illustrations of morphological development of *Bregmaceros neonectabanus*. **A**. Preflexion larva (PKUI 1326, 3.64 mm NL); **B**. Postflexion larva (PKUI 1325, 8.43 mm SL); **C**. Juvenile (PKUI 1323, 12.21 mm SL). Scale bars: 1.000 mm.

**Preflexion larva** (3.64 mm NL). (Fig. 8A; Table 7).

**Table 7.** Measurements and counts of larvae and juveniles of *Bregmaceros neonectabanus*.

**Species**	***Bregmaceros neonectabanus***		
Stage	**Preflexion**	**Postflexion**	**Juvenile**
Number of specimens	1	2	3
**Measurements**			
Total length (mm, TL)	3.80	8.58–9.36	12.35–15.37 (13.63)
Standard length (mm, SL)	3.64	7.90–8.43	11.15–14.11 (12.49)
% in SL			
Head length (mm, HL)	26.17	22.63–22.90	20.07–21.36 (20.54)
Pre-anal length (PaL)	42.16	43.04–46.94	41.09–42.88 (42.25)
Body depth (BD)	26.17	19.08–23.71	14.18–20.14 (17.03)
Head depth (HD)	23.67	18.34–20.96	14.28–18.17 (16.14)
Caudal peduncle depth (CPD)	0	5.66–6.31	6.31–7.25 (6.64)
1^st^ dorsal soft ray length (1DSR)	0	11.84–18.58	18.57–21.56 (20.17)
Interdorsal distance (ID)	0	25.79–26.40	23.68–28.25 (25.66)
Longest pelvic fin ray length	18.98	26.85–35.65	29.58–39.41 (35.80)
% in HL			
Snout length (SnL)	32.59	33.20–37.31	29.85–33.31 (31.39)
Eye diameter	36.60	23.73–25.20	16.94–23.23 (19.75)
**Counts**			
Dorsal-fin rays	10	1–46	1–43~46
Anal-fin rays	17	38	43~48
Pectoral-fin rays	0	17	16~18
Pelvic-fin rays	6	6	6
Caudal-fin rays	4	28	20+10~16

Body laterally compressed, elongate, tapering posteriorly. Abdominal cavity rounded; anus anterior to mid-body. Head large, dorsally convex. Snout steeply inclined; lower jaw projecting beyond upper jaw. Posterior end of the maxilla not reaching the center of pupil. First dorsal fin ray not formed. Pectoral fin as finfold. Pelvic fin bud present immediately posterior to lower jaw; longest ray extending beyond center of abdominal cavity. Anal fin origin posterior to anus; second dorsal fin origin slightly anterior to vertical through anal fin origin. Posterior portions of second dorsal and anal fins forming fin rays. Caudal fin continuous with second dorsal and anal fins; caudal rays forming beneath posterior notochord tip. Melanophores aggregated on dorsal side of abdominal cavity and supraorbital region. No melanophores on the lower side of the operculum and trunk.

**Postflexion larva** (8.43 mm SL). (Fig. 8B; Table 7).

Body elongate, laterally compressed, tapering posteriorly. Abdominal cavity rounded; anus anterior to mid-body. Snout inclined slightly upwards; mouth relatively large. Maxilla slightly extending beyond center of pupil. First dorsal fin ray on the occipital region elongating further. Pelvic fin originating just posterior to lower jaw; longest ray extending beyond anal fin origin. Anal fin origin immediately posterior to anus; second dorsal fin origin vertically above anal fin origin. Anterior and posterior rays of second dorsal and anal fins elongate, with concave mid-region. Melanophores aggregated on head and isthmus. Melanophores on the supraorbital region relatively light. No melanophores on the ventral margin of the operculum. Dorsal side of abdominal cavity densely pigmented. A large dark melanophore on caudal peduncle.

**Juveniles** (11.15–14.11 mm SL). (Fig. 8C; Table 7).

Body long, slightly compressed, tapering posteriorly. Anus anterior to mid-body. Snout slightly inclined upwards. Maxilla reaching or nearly reaching center of pupil. First dorsal fin ray on the occipital region elongating. All fin rays fully formed. Pelvic fin originating just posterior to lower jaw and elongating with growth; longest ray extending well beyond anal fin origin. Second dorsal fin origin slightly anterior to vertical through anal fin origin. Anterior and posterior rays of second dorsal and anal fins markedly longer than middle rays, forming distinct concave profile. Caudal fin rays clearly divided into branched and unbranched rays. Melanophores on head, supraorbital region and dorsal side of abdominal cavity appearing relatively light. A single large melanophore on caudal peduncle.

###### Diagnostic characters.

A single prominent melanophore on caudal peduncle during postflexion and juvenile stages. Melanophores absent on lower part of preopercle.

##### Bregmaceros

Taxon classificationAnimaliaGadiformesBregmacerotidae

sp.

38D98A02-F800-531E-B9D3-569D0CFED647

###### Description.

**Figure 9.**
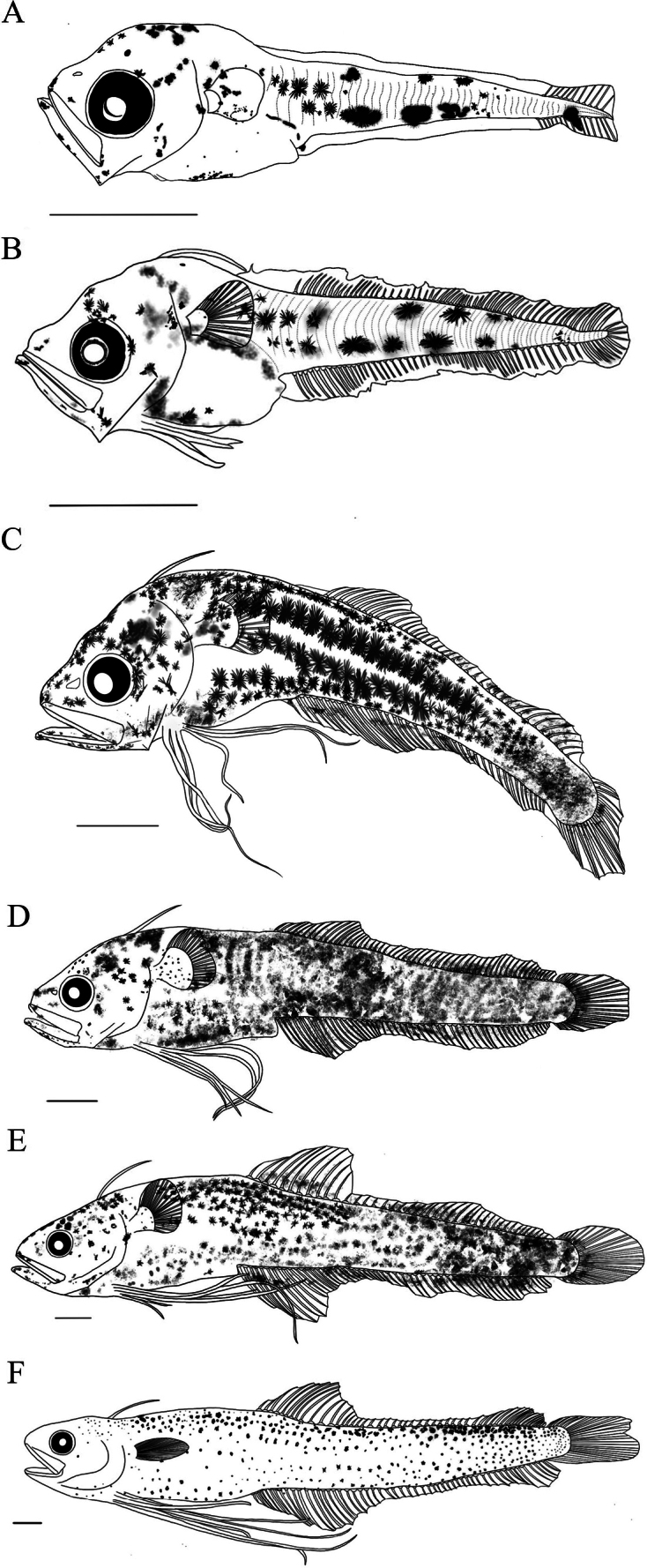
Illustrations of morphological development of *Bregmaceros* sp. **A**. Preflexion larva (PKUI 1349, 3.91 mm SL); **B**. Flexion larva (PKUI 1350, 4.10 mm SL); **C**. Postflexion larva (PKUI 1330, 7.66 mm SL); **D**. Juvenile (PKUI 1339, 11.00 mm SL); **E**. Juvenile (PKUI 1335, 15.92 mm SL); **F**. Juvenile (PKUI 1337, 20.36 mm SL). Scale bars: 1.000 mm.

**Preflexion larvae** (3.91–4.07 mm NL). (Fig. 9A; Table 8).

**Table 8.** Measurements and counts of juvenile of *Bregmaceros* sp.

**Species**	***Bregmaceros* sp**.			
Stage	**Preflexion**	**Flexion**	**Postflexion**	**Juvenile**
Number of specimens	2	1	7	10
**Measurements**				
Total length (mm, TL)	4.01–4.21 (4.11)	4.19	7.65–10.97 (9.61)	12.18–35.48 (17.37)
Standard length (mm, SL)	3.91–4.07 (4.00)	4.11	6.95–10.06 (8.64)	11.00–32.24 (15.75)
% in SL				
Head length (mm, HL)	26.75–30.55 (28.66)	27.69	21.50–25.06 (23.28)	18.50–21.56 (19.33)
Pre-anal length (PaL)	45.36–47.39 (46.38)	44.23	38.78–47.74 (44.80)	38.09–45.84 (40.75)
Body depth (BD)	26.73–28.62 (27.67)	29.19	23.37–26.53 (24.87)	13.14–20.99 (18.56)
Head depth (HD)	28.00–28.67 (28.33)	27.36	19.20–24.10 (21.56)	11.58–19.15 (16.43)
Caudal peduncle depth (CPD)	0	0	7.47–9.51 (8.03)	5.56–7.68 (6.69)
1^st^ dorsal soft ray length (1DSR)	0	16.47	5.66–14.52 (10.74)	11.17–20.81 (14.79)
Interdorsal distance (ID)	0	12.02	20.04–29.49 (25.62)	22.99–28.97 (26.22)
Longest pelvic fin ray length	6.31–6.80 (6.55)	19.89	24.60–40.95 (34.84)	35.35–52.76 (42.95)
% in HL				
Snout length (SnL)	33.77–35.50 (34.64)	33.21	24.46–38.65 (28.76)	21.60–32.70 (28.21)
Eye diameter	33.17–38.45 (35.81)	34.03	19.25–32.95 (26.50)	17.75–27.19 (20.83)
**Counts**				
Dorsal-fin rays	11	1–23	1–39~47	1–47~56
Anal-fin rays	10	38	46~53	53~57
Pectoral-fin rays	0	12	14~20	16~21
Pelvic-fin rays	3	6	6	6
Caudal-fin rays	3	16	18~20+10	20+10~12

Body elongate, laterally compressed, tapering posteriorly. Abdominal cavity rounded; anus anterior to mid-body. Head large, dorsally convex. Snout steeply inclined; lower jaw slightly projecting beyond upper jaw. Posterior end of the maxilla not reaching center of pupil. First dorsal fin ray not formed. Pelvic fin bud present posterior to lower jaw. Pectoral fin as finfold. Anal fin origin immediately posterior to anus; second dorsal fin origin slightly anterior to anal fin origin. Posterior portions of second dorsal and anal fins differentiating into fin rays. Caudal fin continuous with second dorsal and anal fins; caudal rays forming beneath posterior notochord tip. Stellate melanophores dense on top of head; irregularly scattered on snout, lower jaw, anterior tip of upper jaw, postorbital region, and operculum. Dot-like melanophores aggregated on dorsal and ventral surfaces of abdominal cavity. Dark melanophores sporadic on pectoral fin membrane. Large branch-shaped melanophores present on trunk, forming patches along dorsal and ventral sides of the caudal region. A single large melanophore beneath tip of notochord.

**Flexion larvae** (4.11 mm NL). (Fig. 9B; Table 8).

Body compressed, elongate, tapering posteriorly. Head large with shallow dorsal depression. Abdominal cavity rounded; anus anterior to mid-body. Posterior tip of maxilla reaching center of pupil. Snout inclined upwards. Single elongate, separate first dorsal fin ray formed on the occipital region. Pelvic fin elongate, reaching 19.9% of body length. Pectoral fin developing rays. Anal fin origin immediately posterior to anus; second dorsal fin origin anterior to vertical through anal fin origin. Posterior portions of second dorsal and anal fins, with developing fin rays; anterior portions membranous. Branch-shaped melanophores dense on head and above eyes. Melanophores scattered on snout and both jaws. Large melanophores clustered behind operculum and on dorsal and ventral abdominal cavity. Melanophores present on dorsal side of the anus and along pelvic fin rays. Branch-shaped melanophores scattered on trunk, with a series of large branch-shaped melanophores along dorsal and ventral edges of the caudal region. Small branch-shaped melanophores near caudal peduncle.

**Postflexion larvae** (6.95–10.06 mm SL). (Fig. 9C; Table 8).

Body elongate, laterally compressed, tapering posteriorly. Abdominal cavity rounded; anus anterior to mid-body. Snout less steep than in flexion larval stage, but still inclined slightly upwards. Lower jaw projecting slightly beyond upper. Maxilla extending beyond center of pupil but not reaching posterior margin; first dorsal fin ray on the occipital region elongating further. Pelvic fin originating just posterior to lower jaw; longest ray extending beyond anal fin origin. Anal fin origin immediately posterior to anus; second dorsal fin origin vertically above anal fin origin. Second dorsal fin and anal fin rays fully formed; anterior and posterior rays elongate, with concave mid-region. Dark branch-shaped melanophores dense on head, jaws, preopercle, opercle, and supraorbital region. Pectoral fin base and ventral side of abdomen densely pigmented; dorsal abdominal surface unpigmented. Series of large branch-shaped melanophores along trunk, decreasing in size toward caudal peduncle. Scattered melanophores on second dorsal and anal fin membranes, densest at fin bases. Small melanophores clusters around caudal fin base.

**Juveniles** (11.00–32.24 mm SL). (Fig. 9D–F; Table 8).

Body long, slightly compressed, tapering posteriorly. Anus anterior to mid-body. Snout slightly inclined upwards; mouth relatively large. Maxilla reaching or slightly exceeding center of pupil. First dorsal fin ray on the occipital region elongating with growth. All fin rays fully formed. Pectoral fin fan-shaped in smaller juveniles (11.00 mm SL), becoming oval in larger specimens (20.36 mm SL). Pelvic fin originating just posterior to lower jaw and elongating with growth. Longest ray extending well beyond mid-body. Anal fin origin immediately posterior to anus; second dorsal fin origin nearly vertical to anal fin origin. In smaller specimens (11.00 mm SL), anterior and posterior rays of second dorsal and anal fins slightly longer than middle rays; in larger specimens (15.92 mm SL), markedly longer, forming distinct concave profile. Caudal fin rays clearly divided into branched and unbranched rays. Melanophore pattern size-dependent. In smallest juveniles (11.00 mm SL), large dark branch-shaped melanophores covering body, similar to postflexion larval stage. Branch-shaped melanophores scattered on head, snout, anterior tip of jaws, preopercle, operculum, isthmus, and nape. Small dot-like melanophores present at pectoral fin base; abdomen covered with large branch-shaped melanophores. Trunk and caudal region densely pigmented with dark branch-shaped melanophores. Melanophores scattered on second dorsal and anal fin membranes, densest near bases. Caudal peduncle and caudal fin base heavily pigmented. Melanophores becoming smaller with growth, to ~15.92 mm SL. In individuals of ~20.36 mm SL, head melanophores nearly absent; body melanophores transformed from branched to stellate or dot-like, smaller in size. Stellate melanophores forming narrow rows dorsally; ventral melanophores fewer and more widely spaced.

###### Diagnostic characters.

Melanophores distributed throughout body; melanophores becoming denser during larval development from preflexion to postflexion stages, but reduced in size during juvenile stage.

## Discussion

### Possible misidentification in previous studies

Over an 8-year survey (2017–2024) in the northwestern Pacific Ocean, larvae of five species of Bregmacerotidae were identified by comparison of their mtDNA16S rRNA sequences with those of adult voucher specimens, followed by detailed examinations of morphological characteristics across developmental stages. Among these species, one lineage (‘*Bregmaceros* sp.’) superficially resembled *B. atlanticus* morphologically but formed a genetically distinct lineage based on mtDNA sequence comparisons and phylogenetic analyses. These results suggest that the specimens examined in the present study may represent a distinct *Bregmaceros* lineage.

Globally, there have been limited studies on larval stages of bregmacerotid species; one species reported by Mamhot et al. (1992), four species reported by Yoo et al. (1992), and three species reported by Okiyama (2014), revealed several likely cases of misidentification. Notably, *B. neonectabanus* appears to have been misidentified in several previous reports, and the present study provides a detailed larval description that may help distinguish this species from morphologically similar congeners.

The specimen reported as *B. neonectabanus* by Mamhot et al. (1992) was described as possessing melanophores on the top of the head, ventral abdomen, dorsal side of the anus, and lower operculum, as well as along the mid-lateral trunk. In contrast, preflexion larvae of *B. neonectabanus* in the present study lacked melanophores on the head, lower operculum, and ventral abdomen (Fig. 8). The melanophore pattern described by Mamhot et al. (1992) is more consistent with that of *B. nectabanus* preflexion larvae, which exhibit melanophores along the lower operculum and lateral side of the trunk.

Yoo et al. (1992) described larvae of *B. japonicus*, *B. atlanticus*, *B. nectabanus*, and *B. neonectabanus*. Their juvenile *B. japonicus* specimen was reported to lack opercular melanophores and to possess a large melanophore on the caudal peduncle. In contrast, *B. japonicus* juveniles in the present study exhibited dense melanophores across the head, including the operculum (Fig. 6). Moreover, the presence of a prominent melanophore anterior to the caudal peduncle is a key diagnostic feature of *B. neonectabanus*, implying that the *B. japonicus* juvenile reported by Yoo et al. (1992) was likely attributable to *B. neonectabanus*. Similarly, their description of juvenile *B. neonectabanus*, with melanophores scattered on the head, nape, and trunk, and no distinct pre-caudal melanophore, differed markedly from the *B. neonectabanus* specimens examined in this study, which consistently showed little to no head or trunk pigmentation but possessed a distinct melanophore anterior to the caudal peduncle (Fig. 8). This discrepancy implies that *B. neonectabanus* described by Yoo et al. (1992) may represent the *Bregmaceros* sp. in the present study.

Okiyama (2014) described postflexion larvae of *B. japonicus* with large branch-shaped melanophores across the trunk. In contrast, the postflexion larvae of *B. japonicus* examined in the present study showed only small stellate melanophores, suggesting that the specimen described by Okiyama (2014) is more consistent with the *Bregmaceros* sp. in the present study, rather than *B. japonicus* (Fig. 6). Their description of *B. nectabanus*, with the absence of trunk melanophores and a single dorsal row on the abdomen from flexion larval to juvenile stages, was consistent with the present findings. However, the juvenile *B. nectabanus* (26.5 mm SL) described by Okiyama (2014) also exhibited a ventral row of dot-like melanophores above the anal fin base, which was not observed in our juvenile *B. nectabanus* specimens but is characteristic of *B. anchovia* (Fig. 4). Given the high degree of morphological similarity between these species and the later description of *B. anchovia* by Ho et al. (2020b), the juvenile *B. nectabanus* specimens described by Okiyama (2014) may represent *B. anchovia*. Finally, the juvenile *Bregmaceros* sp. described by Okiyama (2014), which exhibits large branch-shaped melanophores across the entire body, appears to be consistent with the *Bregmaceros* sp. in the present study.

### A putative undescribed *Bregmaceros* lineage related to *B. atlanticus*

*Bregmaceros* sp., a putative genetically distinct lineage in the present study showed a genetic distance of 5.93% from *B. atlanticus* based on mtDNA16S rRNA sequences. This result suggests that the specimens may represent a candidate undescribed *Bregmaceros* lineage. Although the partial 16S rRNA fragment was useful for preliminary molecular identification of the collected specimens, the relatively short sequence may be insufficient on its own to support robust species delimitation or definitive phylogenetic conclusions within *Bregmaceros*.

**Figure 10.**
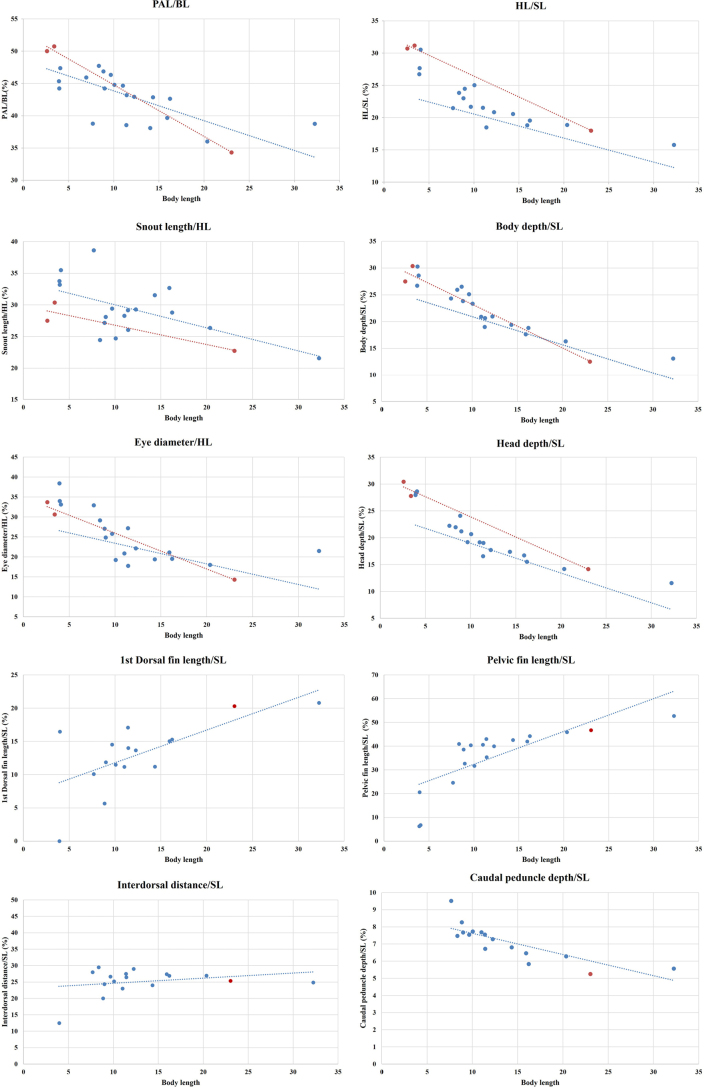
Relative growth of various body parts of larvae and juveniles of ‘*Bregmaceros* sp.’ (blue), *Bregmaceros atlanticus* (red; VIMS 5829, 36811-1, 36811-2).

**Figure 11.**
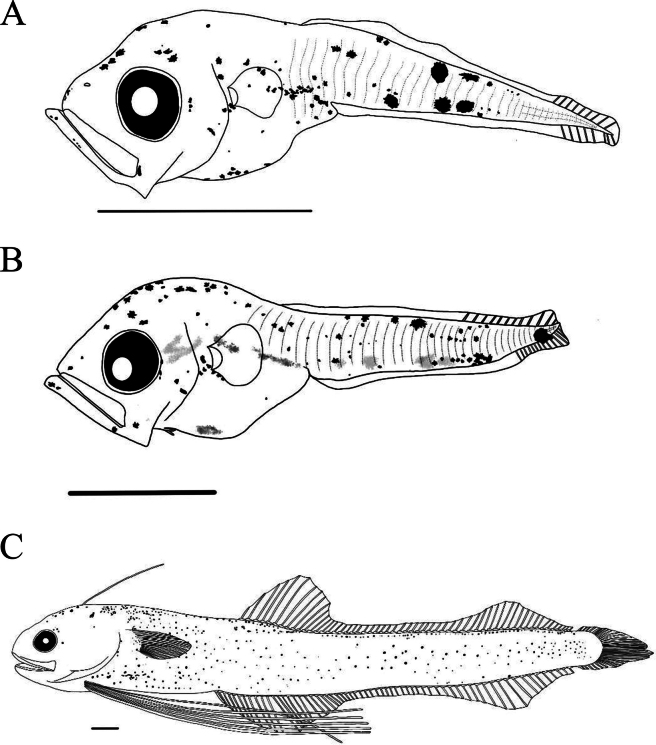
Illustrations of morphological development of *Bregmaceros atlanticus*. **A**. Preflexion larva (VIMS 36811-1, 2.59 mm SL); **B**. Preflexion larva (VIMS 36811-1, 3.40 mm SL); **C**. Juvenile (VIMS 5829, 23.03 mm SL). Scale bars: 1.000 mm.

Larvae identified as this *Bregmaceros* sp. were morphologically identical to those of *B. atlanticus* and to specimens described previously as *Bregmaceros* sp. by Yoo et al. (1992), Lee et al. (2004), and Okiyama (2014). We compared voucher specimens of *B. atlanticus* loaned from the Virginia Institute of Marine Science (VIMS 5829, 20.36 mm SL; VIMS 36811-1, 2.59 mm NL; VIMS 36811-2, 3.40 mm NL) and *Bregmaceros* sp. larvae collected in this study. Meristic and morphometric characters largely overlapped between the two taxa (Fig. 10), and there were no marked differences in fin morphology or counts (Tables 8, 9). Pigmentation patterns were also highly similar between the two taxa. During the larval stage, both *B. atlanticus* and *Bregmaceros* sp. showed large, dark melanophores distributed irregularly along the caudal trunk. In juveniles, these melanophores became smaller and appeared as dot-like or stellate-shaped spots aligned along the lateral trunk. In both taxa, dark melanophores were present on the ventral abdomen, top of the head, base of the pectoral fin, and as a prominent melanophore beneath the posterior end of the notochord in preflexion larvae (Figs 8, 11). Although *B. atlanticus* and *Bregmaceros* sp., a candidate undescribed species closely related to *B. atlanticus* were nearly indistinguishable in external morphology during the larval and juvenile stages, genetic divergence supports the hypothesis that the *Bregmaceros* sp. represents a candidate lineage within *Bregmaceros*.

**Table 9.** Measurements and counts of larvae and juvenile of *Bregmaceros atlanticus* from Atlantic Ocean.

**Species**	***Bregmaceros atlanticus***		
Voucher number	**VIMS 36811–1**	**VIMS 36811–2**	**VIMS 5829**
Stage	Preflexion	Preflexion	Juvenile
**Measurements**			
Total length (mm, TL)	2.73	3.52	24.79
Standard length (mm, SL)	2.59	3.40	23.03
% in SL			
Head length (mm, HL)	30.72	31.20	18.00
Pre-anal length (PaL)	50.04	50.78	34.33
Body depth (BD)	27.53	30.40	12.55
Head depth (HD)	30.45	27.82	14.16
Caudal peduncle depth (CPD)	–	–	5.24
1^st^ dorsal soft ray length (1DSR)	–	–	20.32
Interdorsal distance (ID)	–	–	25.32
Longest pelvic fin ray length	–	–	46.71
% in HL			
Snout length (SnL)	34.00	28.75	22.75
Eye diameter	33.75	30.63	14.30
**Counts**			
Dorsal-fin rays	9	11	56
Anal-fin rays	5	10	58
Pectoral-fin rays	0	0	19
Pelvic-fin rays	3	6	6
Caudal-fin rays	3	16	20+10

Torii et al. (2004) re-examined specimens identified previously as *B. lanceolatus* and, based on morphological differences in opercular branching, the shape of the axillary flap on the shoulder girdle, and the lateral parapophyses of the abdominal vertebrae, described *B. pseudolanceolatus* as a new species. Similarly, as there were no distinct external morphological differences between *B. atlanticus* and *Bregmaceros* sp. during the larval stage, future comparative studies of internal anatomy, such as osteology (Torii et al. 2004; Harold and Baltzegar 2023) and digestive organs (Ho et al. 2020b), are necessary to clarify their taxonomic relationship.

Recent studies in the Mediterranean (Adriatic Sea) and the Red Sea have shown that several specimens previously identified as *B. atlanticus* were in fact *B. nectabanus* (Harold and Golani 2016; Dulčić et al. 2020), implying that the distribution of *B. nectabanus* extends far beyond its previously assumed Indo-Pacific range. Similarly, *B. neonectabanus* has been recorded in the southeastern Atlantic and Indian oceans, indicating a broader geographical distribution than originally reported (Masuda et al. 1986; Harold and Johnson 2016). The type locality of *B. atlanticus* is the Gulf of Mexico, and the species has been reported throughout the Atlantic from the United States and Brazil to West Africa, as well as in the northwestern Pacific, including waters off Korea and Japan (Yoo et al. 1992; Nakabo 2000; Namiki et al. 2007; Fermon et al. 2022). Uncertain distribution of species and widespread misidentifications in the family Bregmacerotidae suggest the presence of a putative undescribed *Bregmaceros* lineage. Therefore, a comprehensive taxonomic revision of the family Bregmacerotidae is required, incorporating external morphology, osteological features, ontogenetic development, and molecular analyses.

### Key to species of early life stage of the family Bregmacerotidae

#### 1. Preflexion larvae

**Table:** 

1	Branch-shaped melanophores present on entire body	***Bregmaceros* sp**.
–	Melanophores absent on the body	**2**
2	Melanophores present on lower part of preopercle	***B. nectabanus***
–	Melanophores absent on lower part of preopercle	***B. neonectabanus***

#### 2. Flexion larvae

**Table:** 

1	Branch-shaped melanophores present on the entire body	***Bregmaceros* sp**.
–	Body largely unpigmented	***B. nectabanus***

#### 3. Postflexion larvae

**Table:** 

1	Melanophores present on the entire body	**2**
–	Body largely unpigmented	**3**
2	Stellate melanophores present on the entire body	***B. japonicus***
–	Branch-shaped melanophores present on the entire body	***Bregmaceros* sp**.
3	A single prominent melanophore on caudal peduncle	***B. neonectabanus***
–	Melanophores absent on caudal peduncle	***B. nectabanus***

#### 4. Juveniles

**Table:** 

1	Melanophores present on the entire body	**2**
–	Body largely unpigmented	**3**
2	Stellate melanophores present on the entire body	***B. japonicus***
–	Branch-shaped melanophores present on the entire body	***Bregmaceros* sp**.
3	Melanophores present in a single row along ventral side of body, snout pointed rather than rounded	***B. anchovia***
–	Melanophores absent on ventral side of body	**4**
4	A single prominent melanophore on caudal peduncle	***B. neonectabanus***
–	Melanophores absent on caudal peduncle	***B. nectabanus***

## Supplementary Material

XML Treatment for Bregmaceros

XML Treatment for Bregmaceros anchovia

XML Treatment for Bregmaceros japonicus

XML Treatment for Bregmaceros nectabanus

XML Treatment for Bregmaceros neonectabanus

XML Treatment for Bregmaceros

## Additional information

### Artificial Intelligence (AI) use

The authors accept full responsibility for the content of the manuscript, including the disclosure of any use of AI.

No AI tools were used in the preparation of this manuscript.

### Author ORCIDs

S.-Y. Koo https://orcid.org/0009-0003-0756-2962

S.H. Myoung https://orcid.org/0000-0002-0630-9565

J.-K. Kim https://orcid.org/0000-0002-8499-406X

### Data availability

All of the data that support the findings of this study are available in the main text or Supplementary Information. 16S rRNA sequences generated in the present study were deposited in the NCBI database under accession numbers PX457420–PX457423 and PX457527–PX457563.

## Supplementary materials

Supplementary material 1 Voucher specimen of Bregmacerotidae species Data type png Explanation note **fig. S1**. Voucher specimen of Bregmacerotidae species. **A, B**. *Bregmaceros neonectabanus*, KAUM-I.104814, KAUM-I.111890; **C–E**. *Bregmaceros atlanticus*, VIMS 5829, VIMS 36811-1, VIMS 36811-2; **F**. *Bregmaceros anchovia*, KPM-NI0062323; **G**. *Bregmaceros pseudolanceolatus*, KPM-NI0048510; **H, I**. *Bregmaceros cantori*, TCWC 19204.01, TCWC 19208.01; **J**. *Bregmaceros lanceolatus*, ASIZP0065753; **K**. *Bregmaceros pseudolanceolatus*, ASIZP0074795. Scale bars: 1.000 mm. https://binary.pensoft.net/file/1713028This dataset is made available under the Open Database License (http://opendatacommons.org/licenses/odbl/1.0/). The Open Database License (ODbL) is a license agreement intended to allow users to freely share, modify, and use this Dataset while maintaining this same freedom for others, provided that the original source and author(s) are credited. Seo-Yeon Koo, Se Hun Myoung, Sang-Chul Yoon, Jung-Hoon Lee, Jin-Koo Kim

## References

[B1] Cohen DM, Inada T, Iwamoto T, Scialabba N (1990) FAO Species Catalogue: An annotated and illustrated catalogue of cods, hakes, grenadiers and other gadiform fishes known to date. FAO Fisheries Synopsis, 442 pp.

[B2] Collins RA, Boykin LM, Cruickshank RH, Armstrong KF (2012) Barcoding’s next top model: an evaluation of nucleotide substitution models for specimen identification. Methods in Ecology and Evolution 3(3): 457–465. 10.1111/j.2041-210x.2011.00176.x

[B3] Darriba D, Taboada GL, Doallo R, Posada D (2012) jModelTest 2: more models, new heuristics and parallel computing. Nature Methods 9(8): 772. 10.1038/nmeth.2109 PMC4594756 22847109

[B4] Dean MM, Houde ED (1984) A new species of Bregmacerotidae (Pisces), *Bregmaceros cantori*, from the western Atlantic Ocean. Bulletin of Marine Science 35(1): 11–19.

[B5] Dulčić J, Bello G, Dragičević B (2020) *Bregmaceros nectabanus* Whitley, 1941 (Teleostei: Bregmacerotidae), a new lessepsian migrant in the Adriatic Sea. BioInvasions Records 9(4): 808–813. 10.3391/bir.2020.9.4.14

[B6] Ekin I (2022) The first fossil record of the codlet *Bregmaceros*? sp. (Thompson, 1840) (Gadiformes, Bregmacerotidae) from the Fırat Formation (Early Miocene-Aquitanian-Burdigalian) of Diyarbakır, Turkey. Journal of the Palaeontological Society of India 67(1): 237–244. 10.1177/0971102320220118

[B7] Fermon Y, Bailly N, Cardiec F, Causse R, Chartrain E, Chirio L, De Bruyne G, Deynat P, Hopkins CD, Lamboj A, Mennesson MI, Mve Beh JH, Paugy D, Sidlauskas B, Sullivan JP, van de Weghe JP, Vigliotta TR, Van Der Zee J (2022) An annotated checklist of the fishes of Gabon. Cybium 46(2–3): 69–317. 10.26028/cybium/2022-462-3-001

[B8] Fricke R, Eschmeyer WN, Van der Laan R (2026) Eschmeyer’s Catalog of Fishes. http://researcharchive.calacademy.org/research/ichthyology/catalog/fishcatmain.asp [accessed 02 June 2026]

[B9] Gill TN (1872) Arrangement of the families of fishes, or classes Pisces, Marsipobranchii, and Leptocardii. Smithsonian Miscellaneous Collections 247: 1–49. 10.5962/bhl.title.18974

[B10] Gill AC, Leis JM (2019) Phylogenetic position of the fish genera *Lobotes*, *Datnioides* and *Hapalogenys*, with a reappraisal of acanthuriform composition and relationships based on adult and larval morphology. Zootaxa 4680(1): 1–81. 10.11646/zootaxa.4680.1.1 31715943

[B11] Hall TA (1999) BioEdit: A user-friendly biological sequence alignment editor and analysis program for Windows 95/98/NT. Nucleic Acids Symposium Series 41: 95–98.

[B12] Harold AS, Baltzegar DA (2023) A new species of *Bregmaceros* (Gadiformes: Bregmacerotidae) from the eastern Pacific Ocean, with comments on *B. atlanticus* and *B. japonicus*. Zootaxa 5352(2): 266–278. 10.11646/zootaxa.5352.2.7 38221449

[B13] Harold AS, Golani D (2016) Occurrence of the Smallscale Codlet, *Bregmaceros nectabanus* in the Mediterranean Sea, previously misidentified as *B. atlanticus* in this region. Marine Biodiversity Records 9(1): 71. 10.1186/s41200-016-0071-0

[B14] Harold AS, Johnson RK (2016) Bregmacerotidae. In: Carpenter KE, De Angelis N (Eds) The living marine resources of the Eastern Central Atlantic Volume 3: Bony fishes part 1 (Elopiformes to Scorpaeniformes). FAO, Rome, 1957–1958.

[B15] Ho HC, Endo H, Chu TW (2020a) A new species of the codlet genus *Bregmaceros* from the western Pacific Ocean (Gadiformes: Bregmacerotidae). Zootaxa 4786(4): 565–573. 10.11646/zootaxa.4786.4.8 33056465

[B16] Ho HC, Endo H, Lee CL, Chu TW (2020b) *Bregmaceros anchovia* sp. nov., a new codlet species from the western Pacific Ocean (Gadiformes: Bregmacerotidae). Zootaxa 4801(3): 559–569. 10.11646/zootaxa.4801.3.8 33056649

[B17] Houde ED (1981) Distribution and abundance of four types of codlet (Pisces: Bregmacerotidae) larvae from the eastern Gulf of Mexico. Biological Oceanography 1(1): 81–104.

[B18] Houde ED (1984) Bregmacerotidae: development and relationships. In: Moser HG, Richards WJ, Cohen DM, Fahay MP, Kendall Jr AW, Richardson SL (Eds) Ontogeny and Systematics of Fishes. American Society of Ichthyologists and Herpetologists Special Publication 1, Lawrence, Kansas, 300–308.

[B19] Ji HS, Yu HJ, Kim JK, Kim DN, Kim ST, Kim JN, Hwang GS (2020) Fish eggs, larvae and juveniles of Korea. Hangeul Graphics, Busan, 442 pp.

[B20] Kang CB, Kim JK, Han KH, Myoung JG (2025) Revised classification and checklist of Korean native fishes. Korean Journal of Ichthyology 37: 398–460. 10.35399/isk.37.4.21

[B21] Kim M, Oh HS, Park SC, Chun J (2014) Towards a taxonomic coherence between average nucleotide identity and 16S rRNA gene sequence similarity for species demarcation of prokaryotes. International Journal of Systematic and Evolutionary Microbiology 64(Pt 2): 346–351. 10.1099/ijs.0.059774-0 24505072

[B22] Kimura M (1980) A simple method for estimating evolutionary rates of base substitutions through comparative studies of nucleotide sequences. Journal of Molecular Evolution 16: 111–120. 10.1007/bf01731581 7463489

[B23] Kochzius M, Seidel C, Antoniou A, Botla SK, Campo D, Cariani A, Garcia Vazquez E, Hauschild J, Hervet C, Hjörleifsdottir S, Hreggvidsson G, Kappel K, Landi M, Magoulas A, Marteinsson V, Nölte M, Planes S, Tinti F, Turan C, Venugopal MN, Weber H, Blohm D (2010) Identifying Fishes through DNA Barcodes and Microarrays. PLOS ONE 5(9): e12620. 10.1371/journal.pone.0012620 PMC2935389 20838643

[B24] Koo SY, Kim JK (2026) First record of *Bregmaceros anchovia* (Bregmacerotidae, Gadiformes) collected from the sea of Jeju-do Island, Korea. Korean Journal of Fisheries and Aquatic Sciences 59: 75–81.

[B25] Koo SY, Myoung SH, Kim JK (2024) Molecular identification and first morphological description of larvae and juveniles of *Neosalanx anderssoni* (Salangidae) collected from the southwestern sea of Korea. Korean Journal of Ichthyology 36(1): 94–100. 10.35399/isk.36.1.11

[B26] Lee EK, Yoo JM, Kim S, Lee TW, Gong YH (2004) Morphological development of larvae and juveniles of codlets *Bregmaceros atlanticus* in the East China Sea. Ocean and Polar Research 26(3): 425–431. 10.4217/opr.2004.26.3.425

[B27] Mamhot JR, Ozawa T, Masuda Y (1992) Occurrence and abundance of Bregmacerotid larvae in Kagoshima Bay, southern Japan, with descriptions of ontogenetic larval characters. Japanese Journal of Ichthyology 39(1): 49–58. 10.1007/bf02905633

[B28] Masuda S, Ozawa T, Tabeta O (1986) *Bregmaceros neonectabanus*, a new species of the family Bregmacerotidae, Gadiformes. Japanese Journal of Ichthyology 32(4): 392–399. 10.1007/bf02905416

[B29] Matsuura Y, Garcia ACDS, Katsuragawa M, Suzuki K (1993) Distribution and abundance of two species of codlet (Teleostei, Bregmacerotidae) larvae from the south-eastern Brazilian Bight. Fisheries Oceanography 2(2): 82–90. 10.1111/j.1365-2419.1993.tb00123.x

[B30] Miller BS, Kendall AW (2009) Early life history of marine fishes. University of California Press, Berkeley, 376 pp. 10.1525/9780520943766

[B31] Motomura H (2020) List of Japan’s all fish species: Current standard Japanese and scientific names of all fish species recorded from Japanese waters. The Kagoshima University Museum, Kagoshima, 560 pp.

[B32] Munro I (1950) Revision of *Bregmaceros* with descriptions of larval stages from Australasia. Proceedings of the Royal Society of Queensland 61(5): 37–53. 10.5962/p.245086

[B33] Nakabo T (2000) Fishes of Japan with pictorial keys to the species. Tokai University Press, Tokyo, 866 pp.

[B34] Namiki C, Teixeira Bonecker A, Salustiano de Castro M (2007) Occurrence and abundance of three larval codlet species (Bregmacerotidae, Teleostei) in the Southwest Atlantic Ocean (12–22°S). Journal of Applied Ichthyology 23(2): 136–141. 10.1111/j.1439-0426.2006.00818.x

[B35] Nelson JS, Grande TC, Wilson MVH (2016) Fishes of the World. 5^th^ edn. John Wiley and Sons, Hoboken, 707 pp. 10.1002/9781119174844

[B36] Okiyama M (2014) An Atlas of the Early Stage Fishes in Japan. Tokai University Press, Tokyo, 1639 pp.

[B37] Palumbi SR (1996) Nucleic acids II: the polymerase chain reaction. In: Hillis DM, Moritz C, Mable BK (Eds) Molecular Systematics, 2^nd^ edn. Sinauer Associates, Sunderland, 205–247.

[B38] Roje DM (2010) Incorporating molecular phylogenetics with larval morphology while mitigating the effects of substitution saturation on phylogeny estimation: A new hypothesis of relationships for the flatfish family Pleuronectidae (Percomorpha: Pleuronectiformes). Molecular Phylogenetics and Evolution 56(2): 586–600. 10.1016/j.ympev.2010.04.036 20434572

[B39] Ryu HJ, Myoung SH, Sohn HS, Kim JK (2024) First morphological description of *Thryssa kammalensis* (Engraulidae, Clupeiformes) larvae and juveniles collected from the southwestern coasts of Korea. Korean Journal of Ichthyology 36(3): 273–281. 10.35399/isk.36.3.7

[B40] Świdnicki J (1991) New data on the osteology of some species of *Bregmaceros* (Teleostei, Gadiformes). Journal of Morphology 208(2): 129–160. 10.1002/jmor.1052080202 29865509

[B41] Tamura K, Stecher G, Kumar S (2021) MEGA11: molecular evolutionary genetics analysis version 11. Molecular Biology and Evolution 38: 3022–3027. 10.1093/molbev/msab120 PMC8233496 33892491

[B42] Tanaka S (1908) Descriptions of eight new species of fishes from Japan. Annotationes Zoologicae Japonenses 7: 27–47. 10.5281/zenodo.15946599

[B43] Thompson JD, Higgins DG, Gibson TJ (1994) CLUSTAL W: improving the sensitivity of progressive multiple sequence alignment through sequence weighting, position-specific gap penalties and weight matrix choice. Nucleic Acids Research 22(22): 4673–4680. 10.1093/nar/22.22.4673 PMC308517 7984417

[B44] Thompson W (1840) On a new genus of fishes from India. Magazine of Natural History 4: 184–187.

[B45] Torii A, Harold AS, Ozawa T, Iwatsuki Y (2003) Redescription of *Bregmaceros mcclellandi* Thompson, 1840 (Gadiformes: Bregmacerotidae). Ichthyological Research 50(2): 129–139. 10.1007/s10228-002-0148-0

[B46] Torii A, Javonillo R, Ozawa T (2004) Reexamination of *Bregmaceros lanceolatus* Shen, 1960 with description of a new species *Bregmaceros pseudolanceolatus* (Gadiformes: Bregmacerotidae). Ichthyological Research 51(2): 106–112. 10.1007/s10228-003-0202-6

[B47] Whitley GP (1941) Ichthyological notes and illustrations. Australian Zoologist 10: 1–50. 10.5281/zenodo.16011384

[B48] Yamaguchi M, Miya M, Okiyama M, Nishida M (2000) Molecular phylogeny and larval morphological diversity of the lanternfish genus *Hygophum* (Teleostei: Myctophidae). Molecular Phylogenetics and Evolution 15(1): 103–114. 10.1006/mpev.1999.0726 10764538

[B49] Yoo JM, Lee EK, Oh BS (1992) Taxonomical study on Bregmacerotidae larvae in the South Sea, Korea. Ocean and Polar Research 14(1): 1–10.

[B50] Zavala-García F, Flores-Coto C (1994) Abundance and distribution of Bregmacerotidae (Pisces) larvae in Campeche Bay, Mexico. Ciencias Marinas 20(2): 219–241. 10.7773/cm.v20i2.960

