# Supplementary figures and images for: New findings on the early life stages of five codlet species (Teleostei, Gadiformes, Bregmacerotidae) from the northwestern Pacific, including a putative undescribed Bregmaceros lineage
Source: Zookeys. 2026 Jul 10;1284:299–328. doi: 10.3897/zookeys.1284.191403 (PMC13379710; doi:10.3897/zookeys.1284.191403)

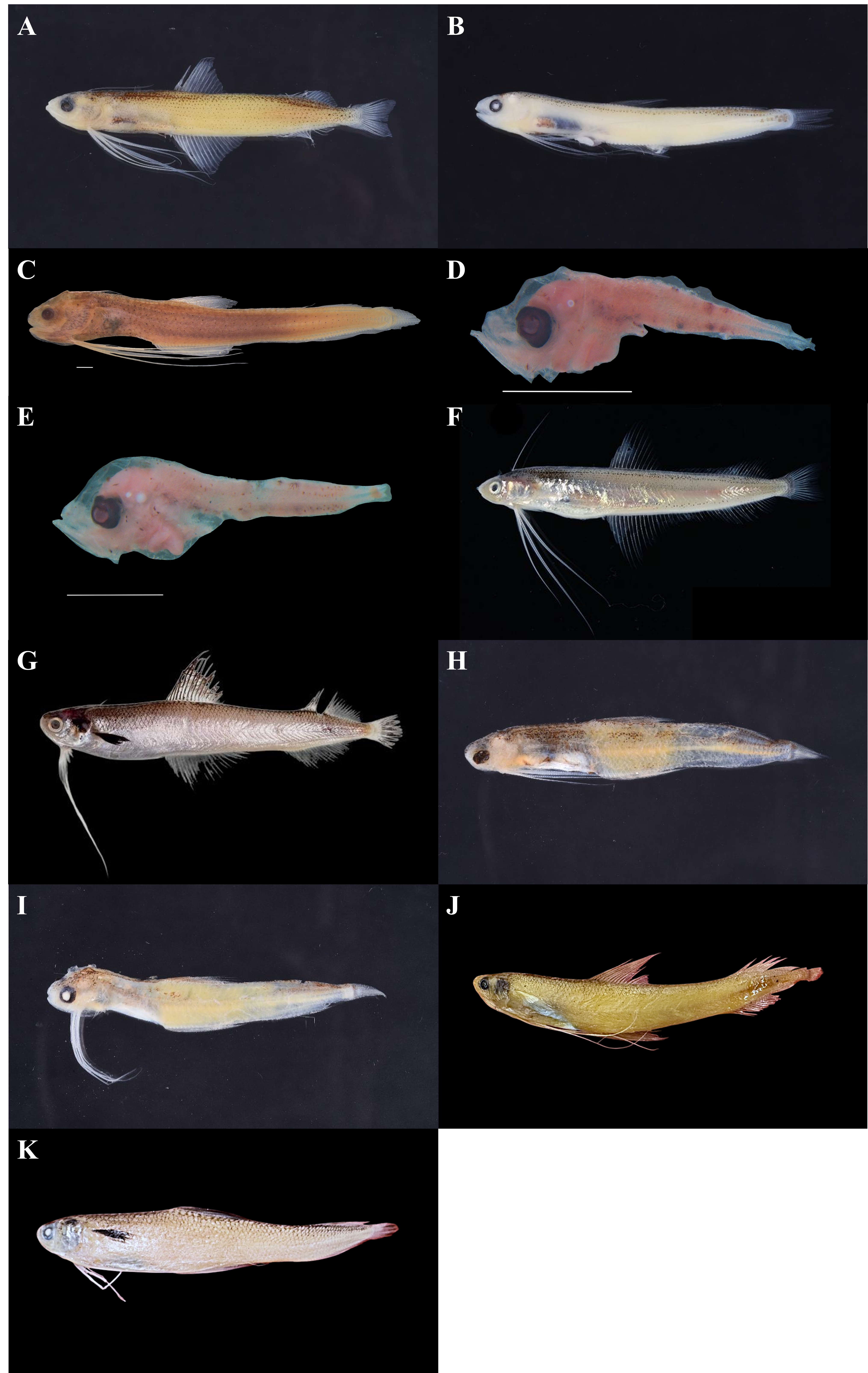

Supplement: Supplementary material 1 — Voucher specimen of Bregmacerotidae species [file zookeys-1284-299_article-191403__-s001.png]
